# Selective Impairment of Rod-Driven Vision in Vitamin A Deficiency: Insights From Examining the Effect of Desensitizing Backgrounds

**DOI:** 10.1167/iovs.66.12.30

**Published:** 2025-09-12

**Authors:** Megan Margetts, Remi Rufus-Toye, Xiaofan Jiang, Shaun M. Leo, Isabelle Chow, Mathura Indusegaran, Pirro G. Hysi, Andrew R. Webster, Christopher J. Hammond, Omar A. Mahroo

**Affiliations:** 1 Physiology, Development & Neuroscience, University of Cambridge, Cambridge, United Kingdom; 2 Institute of Ophthalmology, University College London, London, United Kingdom; 3 Section of Ophthalmology, King's College London, St. Thomas’ Hospital Campus, London, United Kingdom; 4 NIHR Biomedical Research Centre at Moorfields Eye Hospital and the UCL Institute of Ophthalmology, London, United Kingdom

**Keywords:** retina, electroretinography, retinal rod photoreceptor cells

## Abstract

**Purpose:**

The purpose of this study was to explore whether nyctalopia in vitamin A deficiency (VAD) is attributable to simple reduction in quantal catch or to an “equivalent background” phenomenon.

**Methods:**

Five individuals were recruited for experimental electroretinograms (ERGs), including three healthy participants (aged 21 to 47 years), one patient with VAD (aged 70 years), and one patient with *GNAT2*-associated achromatopsia (aged 43 years). Recordings used conductive fiber electrodes and followed dark adaptation and mydriasis. Dim flashes of varying strengths were delivered in the dark to mimic reduction in quantal catch; flashes of fixed strength (0.03 scotopic cd·s/m^2^) were delivered on dim blue backgrounds.

**Results:**

International standard recordings in the patient with VAD showed selective attenuation of dark-adapted responses in severe deficiency, which normalized following treatment. Light-adapted responses did not change. In experimental recordings, both reducing flash strength and applying dim backgrounds reduced ERG amplitude. Reducing flash strength also increased latency of the response, whose rising phase and peak became progressively delayed. Dim backgrounds did not prolong latency. This was seen in all participants, including the patient with achromatopsia, indicating that this was a property of the rod system. In moderate VAD, the dim-flash response was reduced, but not delayed, resembling the response seen in the presence of a dim background.

**Conclusions:**

Our findings indicate that rod system desensitization in VAD likely arises from an “equivalent background” effect, probably arising from activation of phototransduction by free opsin. Activation to a similar degree is known not to occur in cones, helping explain why VAD selectively affects night vision.

Vitamin A deficiency (VAD) is an important cause of preventable childhood blindness, although prevalence has dropped over recent decades.^1^ In developed countries, dietary deficiency is rare, but VAD can also arise from impaired absorption, for example, in the context of prior bowel surgery or liver disease. The chromophore in rod and cone photoreceptors, 11-*cis*-retinal, is derived from vitamin A, hence, deficiency impairs visual sensitivity. With less 11-*cis*-retinal available, a given light stimulus will induce fewer photoisomerizations (referred to as a reduction in “quantal catch”) and elicit a smaller visual signal. Although rods and cones have the same chromophore, one striking feature of the condition is an apparent selective initial impairment of rod function, such that patients complain chiefly of nyctalopia.^2^ Electroretinogram (ERG) responses are consistent with this, showing selective early impairment of rod function, whereas cone-mediated ERGs are relatively spared unless VAD is advanced.^3^

Following photoisomerization to all-*trans*-retinal, 11-*cis*-retinal is regenerated via the retinal pigment epithelium (RPE) in the well-characterized retinoid cycle. Rods and cones both depend on this cycle, but cones can also access an intraretinal visual cycle that involves Muller cells: here, 11-*cis*-retinol is produced, which cones, but not rods, can convert to 11-*cis*-retinal.^4^ This cycle is sometimes invoked as an explanation for the sparing of cones in VAD^3^; the quantal catch may be higher than in rods for a given level of VAD. However, ultimately both cycles should be affected by VAD, and so other mechanisms are likely to contribute to sparing of cone function.

A reduction in 11-*cis*-retinal will not just reduce the quantal catch, but will also lead to an increase in available free opsin. Free opsin itself can activate the phototransduction cascade (causing closure of outer segment cyclic nucleotide-gated channels and hence reduction of the inward cation current, resulting in photoreceptor hyperpolarization) thus leading to visual desensitization.^5^ This has been likened to the effect of the presence of a background, and this “equivalent background” has been used to explain reduced visual sensitivity following a photobleach.^6^^–^^10^ Importantly, this appears to apply to free opsin in rods, but not in cones^11^: in cones, ERG experiments have shown that the inward cation current can be largely preserved even in the presence of a large bleach (with large amounts of opsin unbound to 11-*cis*-retinal).^12^^,^^13^

The selective impairment in rod-mediated vision in VAD is therefore likely to arise from rod desensitization due to the presence of free opsin rather than a reduction in quantal catch. Providing evidence for this in human recordings can be challenging as rod-driven ERG responses are usually undetectable in VAD. In the present study, we recorded ERGs from a patient with VAD in different circumstances, namely when severely deficient, when replete, and when moderately deficient. In the latter context, rod-driven responses were reduced but detectable. We recorded ERGs in healthy individuals, and in the patient when vitamin A replete, in response to dim flashes of different strength delivered in the dark (to mimic the effect of variations in quantal catch), and in the presence of very dim backgrounds (to produce background steady activation of phototransduction), and compared these to the responses obtained in moderate VAD. We also recorded ERGs to similar protocols in a patient with non-functioning cones to check that the phenomena observed arose from the rod system.

## Methods

### Participants

Three healthy participants were recruited for the study (with no ocular pathology other than minor refractive errors). Two patients were also recruited. One patient had VAD (arising from reduced dietary absorption secondary to previous small bowel resections) and underwent international standard full-field ERG recordings^14^ when severely vitamin A deficient (around the time of diagnosis) and also when vitamin A replete following replacement with intramuscular injections. Several months later, her serum vitamin A levels fell to moderately deficient levels, and she underwent ERGs again prior to further intramuscular injections. The second patient recruited for this study had achromatopsia (owing to bi-allelic pathogenic variants in the *GNAT2* gene, which encodes the alpha subunit of the G-protein transducin in cone photoreceptors).^15^^–^^17^

### Ethical Approval

The study had ethical approval and conformed to the tenets of the Declaration of Helsinki. Ethical approval was granted by the NHS research ethics committee (Research Ethics Committee reference number: 11/LO/2029) and all participants gave written informed consent.

### Electroretinogram Recording

Full-field ERGs were recorded with conductive fiber electrodes (Unimed Electrode Supplies, Farnham, Surrey, UK) placed in the lower conjunctival fornix of the eye. Self-adhesive skin electrodes (Unimed Electrode Supplies, Farnham, Surrey, UK) were used as reference and ground electrodes on the temples and forehead, respectively. Recordings were filtered at 0.312 hertz (Hz; high pass) and 300 Hz (low pass), complying with recommended specifications of the International Society for the Clinical Electrophysiology of Vision (ISCEV).^14^ All participants underwent pharmacological mydriasis (with 1% tropicamide and 2.5% phenylephrine) prior to recordings as well as 20 minutes of dark adaptation prior to scotopic recordings.

### Stimuli

Stimuli were full-field and were delivered using the Colordome device (Diagnosys LLC, Lovell, MA, USA) running Espion V6 software (Diagnosys LLC). The patient with VAD underwent ISCEV standard full-field ERG recording protocols as part of her clinical care, before and after vitamin A replacement. When vitamin A replete, she also underwent the experimental protocols described below.

The main test flash corresponded to the ISCEV standard DA 0.01 stimulus: this is a white flash delivered in the dark, with a strength of 0.01 photopic cd·s/m^2^. Although ISCEV stimulus strengths are conventionally expressed in photopic units (which relates to efficiency of cone stimulation), the focus of exploration in the present study is rod signaling, and so stimulus strengths will be expressed in scotopic units. The corresponding scotopic strength of the DA 0.01 stimulus in our equipment was 0.03 scotopic cd·s/m^2^. In healthy participants, ERGs were recorded in response to the test flash and to dimmer and brighter flashes (ranging from 0.001 to 0.2 scotopic cd·s/m^2^), all delivered in the dark. Second, ERGs were recorded in response to the test flash delivered in the presence of very dim backgrounds (ranging from 0.03 to 1 scotopic cd·s/m^2^). Similar stimulus protocols were used when recording from the patient with achromatopsia and from the patient with VAD (after treatment). Finally, the ERG elicited by the test flash delivered in the dark in the patient with VAD when moderately vitamin A deficient was examined and compared with the other responses.

## Results

### ISCEV Standard ERGs in VAD Before and After Vitamin A Replacement

**Figure 1.**
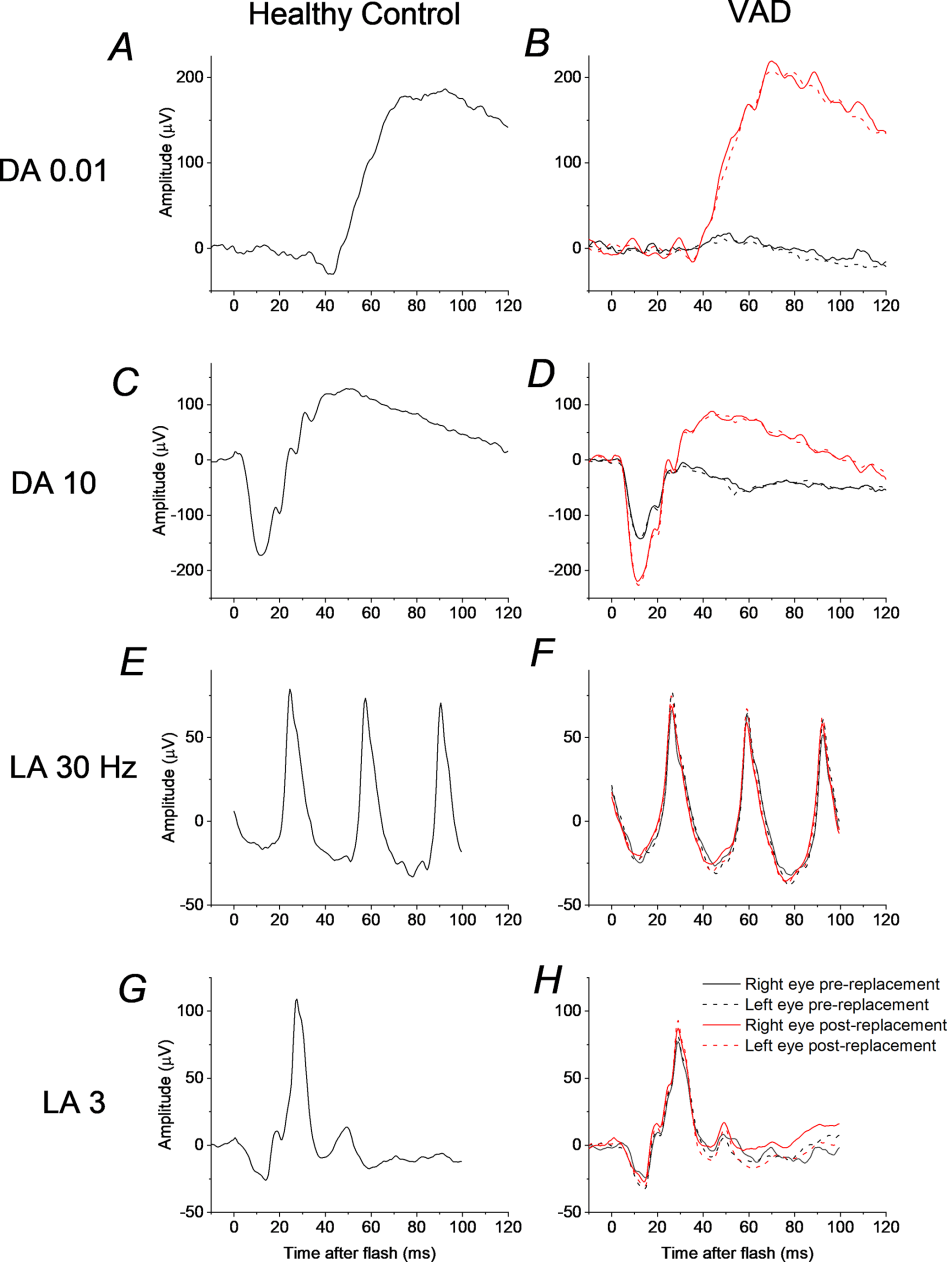
**ISCEV standard ERGs in vitamin A deficiency before and after replacement.** (DA 3 ERGs are not shown, but revealed similar findings to the DA 10 stimulus.) All stimuli are *white*. *Left panels* show example ERGs from a healthy individual. *Right panels* show ERGs from 70-year-old female patient with severe VAD before (*black traces*) and after (*red traces*) vitamin A replacement. (**A,** **B**) ERGs to DA 0.01 stimulus (dark-adapted response to flash delivering 0.01 photopic cd·s/m^2^). (**C,** **D**) ERGs to DA 10 stimulus (dark-adapted response to flash delivering 10 photopic cd·s/m^2^). (**E,** **F**) ERGs to LA 30 Hz stimulus (light-adapted response to 30 Hz flickering stimulus delivering 3.0 photopic cd·s/m^2^ in the presence of *white background* of 30 photopic cd/m^2^). (**G,** **H**) ERGs to LA 3 stimulus (light-adapted response to flashes delivering 3.0 photopic cd·s/m^2^ in the presence of *white background* of 30 photopic cd/m^2^). All traces are averages of multiple stimulus presentations.

Figure 1 shows dark-adapted and light-adapted full-field ERGs elicited by ISCEV standard stimuli. The left panels show responses from a healthy individual for reference; the righthand panels show responses from the patient with VAD recorded when severely deficient in vitamin A (black traces) and following normalization of serum vitamin A levels (red traces) following intramuscular replacement. The patient was a 70-year-old woman who complained of progressive problems with vision in low light levels over the prior several months. Her medical history was significant for Crohn's disease and multiple small bowel resections. She was found to have very low vitamin A levels (0.19 micromol/L [lower limit of reference range = 1.04 micromol/L]). She underwent 3 intramuscular injections of vitamin A and after 2 weeks she felt her vision had normalized. The red traces show ERGs obtained when vitamin A replete (2.7 micromol/L). The ERGs confirm that her dark-adapted (rod-driven) responses were abnormal when vitamin A deficient, whereas her light-adapted (cone-driven) responses were normal; following vitamin A replacement dark-adapted responses normalized, and the light-adapted ERGs were unchanged.

Because dark-adapted mixed and strong flash (DA 3 and DA 10) ERGs contain cone-driven signals in addition to rod-driven responses, the remainder of the investigation focused on dim-flash responses, which are expected to contain minimal cone-derived signals.

### Dim-Flash ERGs in the Dark and on Dim Backgrounds in Healthy Participants

**Figure 2.**
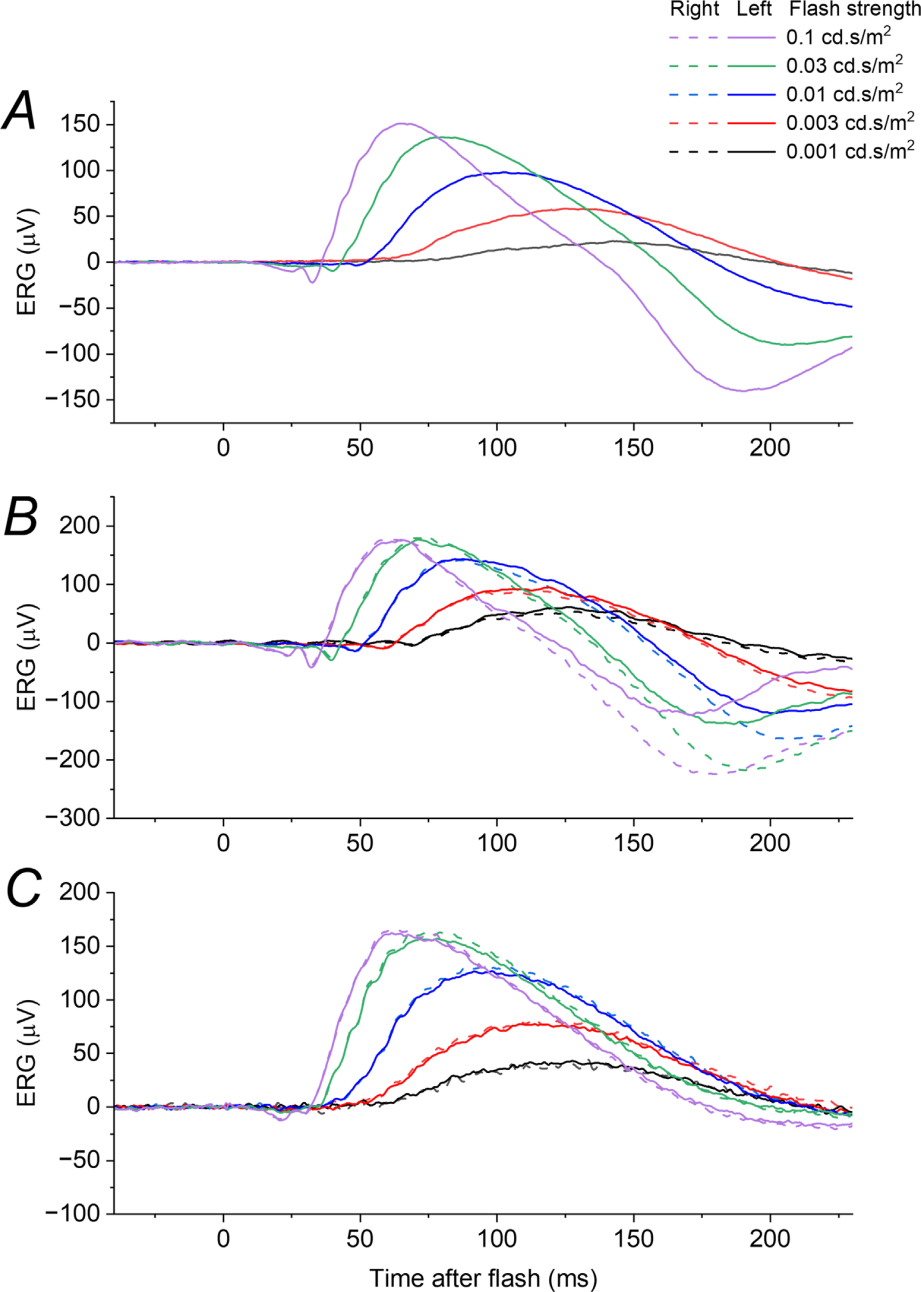
**ERGs elicited by very dim flashes delivered in the dark in healthy participants.** Each panel shows dark-adapted ERGs to flashes of a range of strengths; each trace is the average of multiple flash presentations. The *green traces* in each panel correspond to the ISCEV standard DA 0.01 stimulus. (**A**) ERGs from a 47-year-old man (recordings only undertaken in the left eye). (**B**) ERGs from a 20-year-old woman. (**C**) ERGs from a 29-year-old man. In all cases, as flash strength decreases, the response latency appears to increase, with the initial rise of the b-wave and the subsequent peak occurring at progressively later post-flash times.

Figure 2 depicts ERGs recorded in response to dim flashes of varying strength delivered in the dark, from three healthy participants. For the dimmest flashes, a b-wave is clearly seen. As flash strength increases, the b-wave amplitude increases and the peak also becomes earlier. For the stronger flashes, an a-wave emerges. The green trace in all panels shows the response to the DA 0.01 stimulus, which had a corresponding scotopic strength of 0.03 cd·s/m^2^.

**Figure 3.**
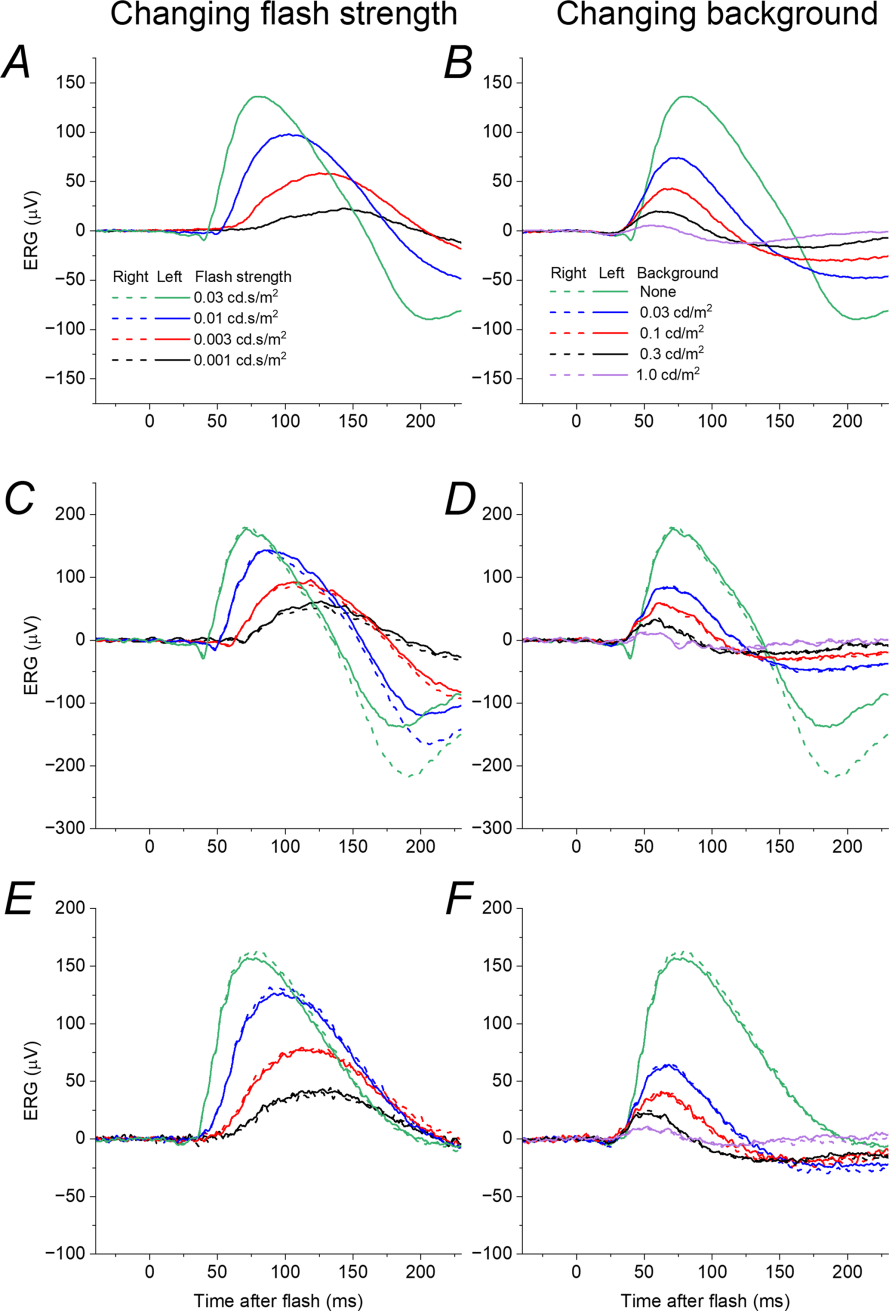
**Effect of varying flash or background strength on dim-flash ERGs in healthy participants.** *Left panels* show ERGs elicited by dim flashes of varying strength: these are replotted from Figure 2, omitting flash strengths greater than the test flash (0.03 cd·s/m^2^). *Right panels* show ERGs in the same participants in response to the test flash delivered in the dark or on very dim backgrounds of varying strength (up to 1 cd/m^2^). (**A, B**) ERGs from a 47-year-old man (recordings only undertaken in the left eye). (**C, D**) ERGs from a 20-year-old woman. (**E, F**) ERGs from a 29-year-old man. As background strength increases (*right panels*) the response rises at a similar time and peaks earlier (in contrast to the delayed rise and later peak seen in the *left panels*).

In Figure 3, lefthand panels plot the responses seen in Figure 2, but for flash strengths up to 0.03 cd·s/m^2^. The right panels plot, with the same scale, responses to the 0.03 cd·s/m^2^ flash, but delivered in the presence of dim backgrounds of increasing strength. The panels on the left mimic the effect of a reduction in quantal catch; the panels on the right show the effect of a background. Both lead to a reduction in response amplitude, but the form of the responses are quite different: in the dark, reducing the flash strength leads to a smaller amplitude response which begins later and reaches a peak at a later time; the effect of a background also produces a smaller amplitude response, but the waveform begins at a similar post-flash time (or slightly earlier) and does not have a prolonged peak time (the peak time appears shortened).

An earlier b-wave peak time is a known feature of cone-driven responses. Backgrounds lead to desensitization of the rod system, and so it is possible that the features observed in the righthand panels of Figure 3 emerge from unmasking cone-driven contributions rather than being features of the rod system. To explore this, we used the same experimental protocol in a patient with achromatopsia, a condition in which cones are non-functional.

### Dim-Flash ERGs in Achromatopsia

**Figure 4.**
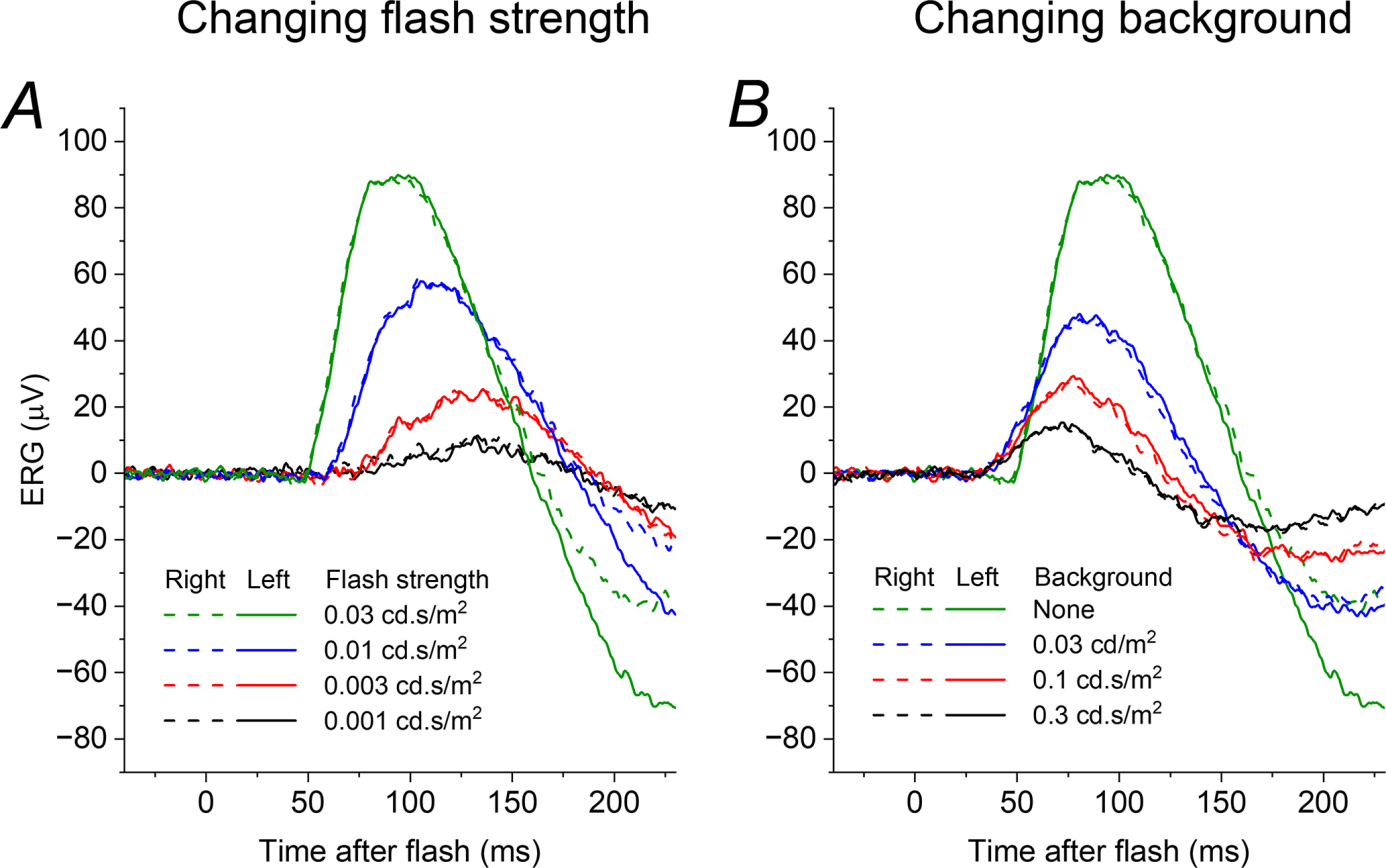
**Effect of varying flash or background strength on dim-flash ERGs in a patient with molecularly proven achromatopsia.** The patient is a 43-year-old man with bi-allelic pathogenic variants in *GNAT2*. (**A**) ERGs in response to dim flashes of varying strength in the dark. (**B**) ERGs elicited by a flash of constant strength (0.03 cd.s/m2) delivered in the dark and on different strengths of dim background. The ERGs show similar features to those seen in the healthy participants (Figs. 2, 3), indicating that these features arise in the rod system.

Figure 4 shows ERGs obtained in response to the same protocol as shown in Figure 3, recorded from a patient with molecularly proven *GNAT2*-associated achromatopsia. The same features are seen as in the healthy participants, indicating that they arise from the rod system and not from cone-driven signals (the patient has absent cone function).

### Dim-Flash Responses in Moderate Vitamin A Deficiency

**Figure 5.**
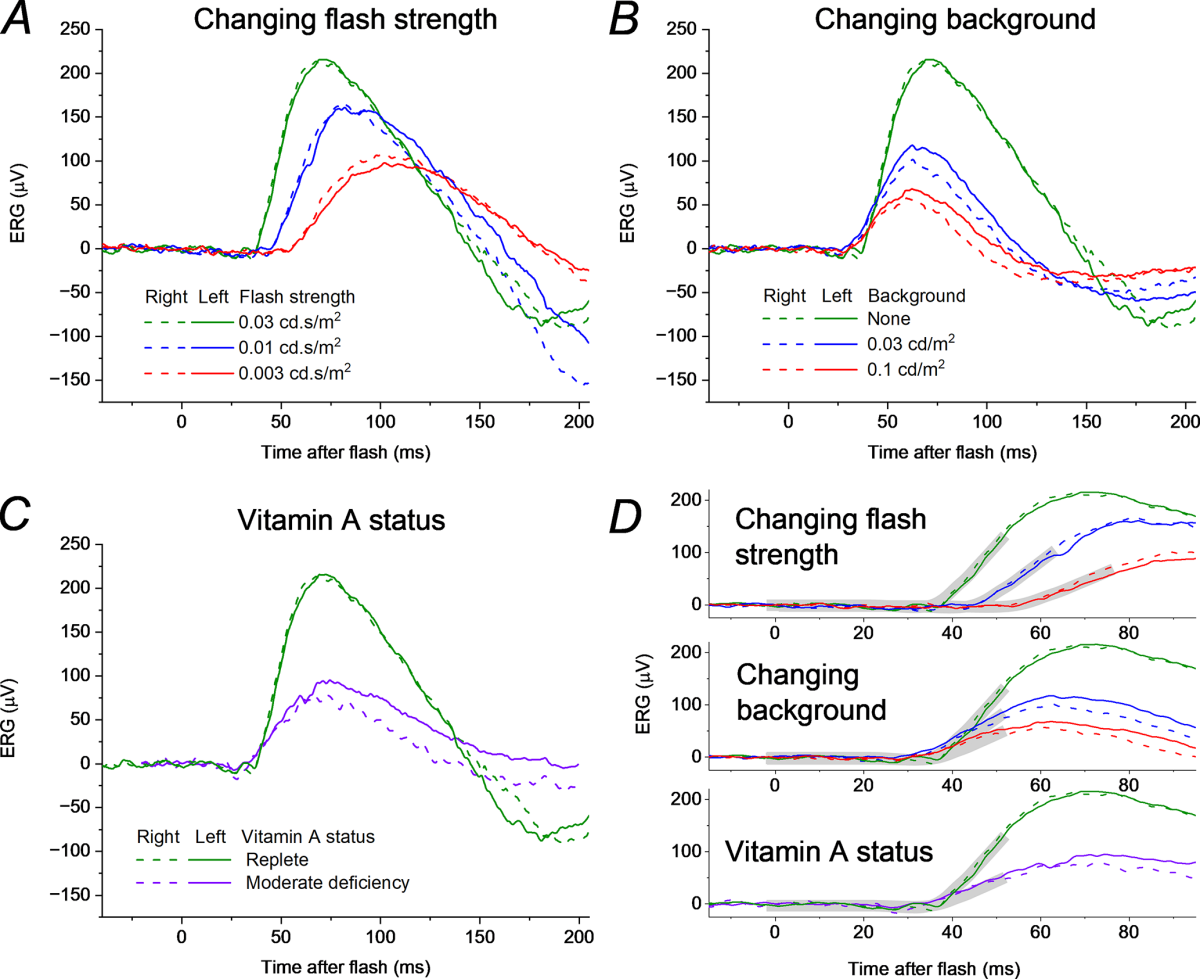
**Dim-flash ERGs in** **a patient with** **VAD** **when replete and moderately deficient.** **(A,** **B**) ERGs recorded in response to varying strengths of dim flash delivered in the dark (**A**) and to a flash of constant strength delivered on dim backgrounds of different strength (**B**), in a 70-year-old woman with normal vitamin A levels (2.7 micromol/L) following replacement after prior deficiency. ERGs show the same features seen in the other participants (Figs. 3, 4). (**C**) ERGs recorded to the test flash delivered in the dark in the setting of subsequent moderate vitamin A deficiency (0.58 micromol/L; *purple traces*). ERGs elicited by the same flash when vitamin A replete (*green traces*) are also shown for comparison. The ERG in vitamin A deficiency resembles the effect of a background (**B**) rather than the response to a dimmer flash (**A**). (**D**) ERGs in the three contexts plotted on an expanded time scale to show the rising phase of the response: this is progressively delayed for dimmer flashes, but occurs at the same post-flash time on the backgrounds and in vitamin A deficiency. *Grey curves* highlight the rising phase of the responses.

Figure 5 shows responses from the patient with VAD (whose ISCEV standard ERGs were plotted in Figure 1). Panels A and B show ERGs obtained when the patient's vitamin A levels were within the normal range (following replacement). These were elicited by the same protocols shown in Figures 3 and 4 and show the same features: weaker flashes give smaller amplitude ERGs which show greater latency, starting to rise at later post-flash times and peaking later; the effect of increasing strength of dim background also leads to smaller amplitudes, but the responses have a similar (or shortened) latency. Panel C plots the response to the 0.03 cd·s/m^2^ test flash (the DA 0.01 stimulus) when the patient had moderate vitamin A deficiency (the response recorded when replete is also plotted in the same panel for comparison). The form of the response resembles those obtained in the presence of dim backgrounds (panel B) rather than the response to dimmer flashes (panel A). Panel D replots the responses from panels A to C, but on an expanded time scale to show the initial rising phase with greater clarity (highlighted by the grey curves): reducing flash strength in the dark leads to a response with greater latency, beginning to rise at later post-flash times; however, responses on stronger backgrounds, and in moderate vitamin A deficiency, appear to begin to rise at the same (or even earlier) post-flash time. These findings are consistent with the mechanism for reduced rod system sensitivity in VAD arising from an “equivalent background”-like effect rather than a direct effect of reduction in quantal catch.

## Discussion

The present study explored, using the ERG, the hypothesis that the diminished rod system sensitivity in VAD could be explained by an “equivalent background” phenomenon (likely owing to persistent activation of the phototransduction cascade by free opsin) rather than directly by the reduction in quantal catch. In VAD, rod-driven ERGs are frequently undetectable, but, here, we additionally undertook recordings in a state of moderate deficiency to allow investigation of the form of rod-driven responses. The experimental protocol interrogated the effect on dim-flash responses of varying flash strength and background strength, finding the same effects in all participants. Responses in moderate VAD mimicked the effect of a background rather than a reduction in flash strength.

The effect of backgrounds and bleaching on the rod dim-flash ERG b-wave in healthy individuals was investigated in detail by Cameron et al.,^18^ and the findings of the present study agree with their findings: the b-wave peaks earlier as background strength increases. In that study, blue flashes were used, whereas in the present study, white flashes were used for greater agreement with international standards: the test flash selected for the current study was identical to the ISCEV DA 0.01 stimulus. The cone-driven b-wave elicited by dim flashes in the dark-adapted state (also termed the “x-wave”) is known to have a markedly shorter peak time compared to the rod-driven component.^19^ Thus, the question can arise as to whether the accelerated peak time seen in the dim-flash responses on dim backgrounds actually reflects a cone-driven component. This possibility was investigated in the present study by using the same experimental protocol in a patient with absent cone function: the same kinetics were observed, indicating that such features arise from the rod system.

The dark-adapted dim-flash b-wave arises from rod bipolar cell signals. In the present study, this was used to infer changes at the level of the photoreceptor. Investigating the ERG a-wave might in theory provide a more direct way of assessing photoreceptor signals. However, the flash strength required to elicit a reproducible a-wave is likely to also generate a cone-driven response, with cone photoreceptors (and cone-driven OFF-bipolar cells) also contributing to the recorded a-wave. There are various methods in the literature for quantifying the dark-adapted cone system contribution to the a-wave, so that this can be “subtracted out.” These include delivering photopically matched red flashes, delivering identical flashes immediately (approximately 1 second) after a very strong flash (at a time when cones, but not rods, will have recovered) or delivering similar flashes on a rod-saturating background.^20^^–^^27^ However, all of these protocols have limitations, including imperfect estimation of the dark-adapted cone system response and, in some cases, increased testing burden for the subject (in terms of duration and comfort). If a rare situation arose in which an individual with achromatopsia developed VAD, then ERG a-wave analysis could provide a more direct way of interrogating the effect on rod photoreceptor sensitivity.

Our data show the different effects of steady cascade activation and reduction in quantal catch on the kinetics of the international standard DA 0.01 ERG, and also highlight the potential utility of focusing on the timing of the commencement of the rising phase of the response (this is not one of the parameters conventionally measured in standard recordings). It is possible that such analyses will yield insight into mechanisms of impairment in other diseases affecting rod photoreceptor signals.
